# Sol-Gel Thin Films for Plasmonic Gas Sensors

**DOI:** 10.3390/s150716910

**Published:** 2015-07-13

**Authors:** Enrico Della Gaspera, Alessandro Martucci

**Affiliations:** 1 CSIRO Manufacturing Flagship, Bayview Ave, Clayton, Victoria 3168, Australia; E-Mail: enrico.dellagaspera@csiro.au; 2 Dipartimento di Ingegneria Industriale ,Universita’ di Padova, Via Marzolo 9, Padova 35131, Italy

**Keywords:** surface plasmon resonance, nanoparticles, optical sensors, metal oxide, TiO_2_, ZnO, NiO, Au, Ag

## Abstract

Plasmonic gas sensors are optical sensors that use localized surface plasmons or extended surface plasmons as transducing platform. Surface plasmons are very sensitive to dielectric variations of the environment or to electron exchange, and these effects have been exploited for the realization of sensitive gas sensors. In this paper, we review our research work of the last few years on the synthesis and the gas sensing properties of sol-gel based nanomaterials for plasmonic sensors.

## 1. Introduction

Dangerous gas detection has become in the last years a challenging task for several applications, first of all in the safety of working and living environments: in fact, toxic gases like CO or volatile organic compounds (VOCs) like formaldehyde are commonly found in these locations, the former coming from insufficient oxygen content in the combustion of coal or gases for heating devices, the latter coming from indoor furniture, because it is commonly used in combination with urea, melamine or phenol to obtain thermosetting resins which are used in coatings, adhesives and foams. Another key topic associated to sensors is related to the so called comfort applications, for example in air monitoring inside buildings or cars, where the target gas may not be highly hazardous or toxic, but its detection and elimination from the environment can improve the air quality. Other applications can be found in chemical plants, inside reactors, pipes, furnaces, where the reacting atmosphere has to be checked in order to keep the overall process inside standard parameters, or where the exhaust gases have to be purified from dangerous compounds before being discharged in the environment.

According to the IUPAC (International Union of Pure and Applied Chemistry), “a chemical sensor is a device that transforms chemical information, ranging from the concentration of a specific sample component to total composition analysis, into an analytically useful signal. The chemical information, mentioned above, may originate from a chemical reaction of the analyte or from a physical property of the system investigated” [1].

Sensors are comprised of two main parts: a receptor that transforms a chemical information into a measurable form of energy, and a transducer, that transforms this energy into a signal. If the receptor reacts with the analyte, the sensor is defined a “chemical sensor”; if this reaction involves biochemical species the sensor is usually described as a biochemical sensor. If no chemical reaction takes place and the effect of the interaction of the analyte with the receptor is the change of some of its physical properties (mass, temperature, electrical, magnetic, optical properties), the sensor is said to be a physical sensor. 

There is a variety of sensing devices that rely on different chemical or physical phenomena: in fact, the analyte presence can be detected through changes in electrical properties (conductivity, impedance, capacitance), optical properties (absorption, reflection, luminescence, refractive index) or other physical properties (mass, thermal conductivity, acoustic waves propagation), measuring the reaction heat, analyzing specific electrochemical or biochemical recognition, and so on.

Among the different transducing platforms, optical sensors have attracted a lot of interest in the last decades since they enable to widen the range of operative parameters compared to electrical sensors: in fact, variation in intensity, frequency, polarization and phase of the transmitted/reflected light can be analyzed, and this can in principle improve the device performances by lowering the cross sensitivity between different gases. Moreover, they are electromagnetic noise independent, they don’t require contact measurements; eventually they can be implemented in optical fiber devices allowing fast and easy signal transport and *in situ* measurements with a compact, flexible and environmental robust setup [2].

As described in [3], optical chemical sensors can be divided in two main categories: direct optical sensor, in which the analyte is detected directly monitoring some intrinsic optical property, such as a specific absorption band of a gas molecule, or reagent-mediated sensors, in which the analyte is detected after its interaction with a sensitive material. In the second case, it is possible to distinguish three main optical gas sensors: absorbance-based, luminescence-based and surface plasmon resonance (SPR)-based, according to the different principles of gas detection. The absorbance-based sensors employ variation in optical absorption, transmission or reflection after the interaction of the light with the active materials in the presence of a target molecule.

In the luminescence-based sensors, the photo-luminescence or chemo-luminescence properties of the sensing material are monitored and related to the target gas concentration: the setup is basically the same as absorbance-based sensors, with the exception of the photoluminescence based devicesthat require the presence of an excitation source to promote the luminescence properties of the material.

In the present paper, we will focus our attention on the SPR-based sensors.

Materials that possess a negative real and a small positive imaginary dielectric constant (like metals) are capable of supporting an SPR. This resonance is a coherent oscillation of the surface conduction electrons excited by an electromagnetic radiation [4]. When a surface plasmon is confined to a structure of a size comparable to the wavelength of the incoming light (for example, like in the case of Au or Ag nanoparticles), the metal free electrons participate in the collective oscillation, and a localized surface plasmon resonance (LSPR) is excited. The excitation of the localized surface plasmon results in a strong light scattering, in the appearance of an intense localized surface plasmon absorption band and in the enhancement of the local electromagnetic field. The frequency and intensity of the LSPR peak highly depends on the geometry and distribution of the metallic nanostructures, and on the properties of the dielectric surrounding them. 

The LSPR peak of Au and Ag nanoparticles (NPs) is sensitive to both dielectric constant changes in the supporting matrix and to changes in electron density of the metal NPs [5,6] and both these effects have been exploited for gas sensing applications. For example, in their seminal work, Ando *et al.,* presented optical sensors for CO and H_2_ based on Au NPs dispersed in metal oxide matrices using a variety of techniques including sputtering, pyrolysis and precipitation methods. Comparing the response of the pure metal oxides with respect to the Au-containing films, the authors observed a wavelength dependent response around the Au LSPR range, which enabled the authors to discriminate between the two target gases but also observe a catalytic effect of Au in forming carbonate species promoting CO oxidation to CO_2_ [7,8].

Another example of discrimination of different gaseous species using optical spectroscopy on Au-containing metal oxide films comes from Carpenter and coworkers, who first observed a blue shift of LSPR peak of Au when Yttria-Stabilized Zirconia (YSZ) films containing Au NPs were exposed to CO [9] and then observed a different spectral change of similar films when exposed to NO_2_ and O_2_ [10]. Eventually, the authors used spectral multivariate analysis to analyze the changes in Au LSPR and were able to optically discriminate between CO, H_2_ and NO_2_ using a CeO_2_-Au nanocomposite [11]. Often, optical shifts in Au NPs/metal oxide gas sensors are considered to be the consequence of both electrons exchange on the metal at the metal/oxide interface as well as to dielectric changes in the support. Metal nanostructures exhibiting an LSPR have also been used for ultra-sensitive detection of refractive index changes: for example, Van Duyne showed that triangular Ag NP arrays fabricated by nanosphere lithography could be used to detect different inert gases (N_2_, Ar, He).Even if their refractive index is extremely similar, it is different enough to cause variation in the LSPR frequency that can be optically monitored [6]. Moreover, the sensitivity could be increased even further by embedding the plasmonic NPs within a highly porous matrix [12]. Plasmonic nanoparticles can also be used to “sense” refractive index changes due to chemical reactions: for example, Au antennas have been put in contact with [13] or in close proximity to [14,15] palladium and the hydrogenation of Pd to PdH_x_ when exposed to H_2_ gas can be monitored by observing the changes in the LSPR resonance of Au NPs induced by the variation in refractive index between Pd and PdH_x_. For further reading, extensive reviews have been published recently [4,16,17].

Surface plasmons can be excited also at the interface of metal-dielectric with dimension much larger than the wavelength of the incoming light (for example like in the case of Au film deposited on a glass substrate). In this case, the electromagnetic wave coupled to the collective electron oscillations and propagating at the metal-dielectric interface, is called surface plasmon polariton (SPP) or propagating plasmon. In the case of SPP, plasmons propagate in the *x*- and *y*-directions along the metal-dielectric interface for distances on the order of tens to hundreds of microns, and decay evanescently in the *z*-direction with 1/e decay lengths on the order of 200 nm [18,19].

Since these coherent oscillations of metal surface electrons are extremely sensitive to changes in the dielectric properties of the interfacing material (for example the refractive index), this property can be exploited for developing sensors with high sensitivity [20,21]. The theoretical basis of the phenomenon was laid by Wood [22] at the beginning of the twentieth century, analyzing the anomalous light diffraction on diffraction gratings, but real progress was made when Ritchie [23] theoretically demonstrated the existence of SPP and Otto [24] and Kretschmann [25] described the attenuated total reflection (ATR) prism coupling scheme for SPP excitation.

The conditions for SPP propagation depend on the properties of the metal film, the wavelength of the incident light and the refractive index of the media on both side of the film. The propagation constant *β* of the surface plasma wave propagating at the interface between a semi-infinite dielectric and metal is given by the following expression:

(1) $$\beta =k\sqrt{\frac{\varepsilon_{m}n_{s}^{2}}{\varepsilon_{m}+n_{s}^{2}}}$$

where *k* denotes the free space wave number, *ε_m_*, the dielectric constant of the metal, and *n_s_* the refractive index of the dielectric. As may be concluded from Equation (1), the surface plasmon wave may be supported by the structure providing that *ε_1m_* < −*n_s_^2^*. Metals that satisfy this requirement are, for example, silver, gold, copper, aluminum, sodium, and indium [26]. Among them, gold and silver are preferable, since indium is quite expensive, sodium too reactive, copper and aluminum too broad in their SPR response; gold is the most used thanks to its high resistance to oxidation compared to silver, even if a silver film may yield a more distinct SPR spectrum with respect to gold.

In most common practical applications, the metal film is deposited onto the base of a glass prism (Kretschmann configuration); from a certain angle of incidence, the incoming light is totally reflected, and an evanescent wave penetrates through the metal film. This electromagnetic field is called evanescent wave because the amplitude of the wave decreases exponentially with increasing distance from the interface surface, decaying over a distance of about the light wavelength [27]. As a result of this evanescent wave formation, a decrease of intensity of the reflected light is observed. 

The glass prism can also be replaced by a diffraction grating: for a proper combination of the period of the grating, the angle and wavelength of the incident wave, a surface plasmon is generated [28].

Sensors based on propagating plasmons are intrinsically non-selective, because every analyte that can cause a variation in the electric-dielectric properties at the interface with the metal layer is, in principle, capable of altering the plasmon propagation so the specificity of these sensors is usually achieved by modifying the metal surface with a functional layer to allow specific interaction with the target analyte. This strategy has been successfully applied for chemosensors and extensive reviewson the topic have been published [29,30], highlighting also the possible different configurations of SPR sensors, including the attractive implementation within optical fibers.

It is important to underline that, in the case of reagent-mediated optical sensors, very often the sensitive material is a thin film (such as a polymer or a metal oxide), which reacts with the target gas and the changes in its properties (chemical, electrical, *etc.*) are monitored using plasmonic probes, either looking at the LSPR of metal NPs or analyzing the variation in propagating plasmons at the interface of metal film-dielectric.

Thin films may be obtained by several different techniques, such as sputtering, chemical vapor deposition, and sol-gel. Sol-gel is certainly among those preparation methods giving the highest level of control for all the above parameters, as it allows one to obtain any combination of oxides by perfectly mixing all the components at molecular level, to obtain crystalline NPs with controlled size and shape, to get nanoporous materials with good control of size and connectivity of pores. 

Sol-gel is relatively simple to transfer to an industrial scale and is particularly suitable for the deposition of thin films, as it is based on the deposition of a thin layer of the liquid precursor solution over a substrate at room temperature (by dip-coating, spin-coating, spraying techniques), followed by a thermal treatment. In the case of thin films, where the cost of precursors is minimized, the method is also not expensive. For these reasons sol-gel may offer interesting challenges in the field of gas sensors, and indeed it is actually largely investigated. 

An example of the potentiality of the sol-gel method is offered by the synthesis of nanocomposite thin films constituted of a nanoporous amorphous silica matrix and homogeneously distributed semiconducting metal oxide NPs. The nanoporosity provides the path for gas molecules to reach all the volume of the sensing material, and the possibility for the NPs to be efficiently exposed to the analyte [31].

The sol-gel technique has been widely used to prepare functional materials for gas sensing applications [32,33]. In particular, LSPR sensors based on sol-gel film has been extensively studied by Ohodnicki and co-workers. They developed different metal oxide sol-gel matrices (SiO_2_, TiO_2_, ZrO_2_ and YSZ) containing Au NPs for CO and H_2_ sensors operating at high temperatures (up to 1000 °C) [34,35,36]. The sol-gel film has been implemented on optical fibers for the simultaneous gas and temperature sensing [35]. Sol-gel film has been also used as a sensitive layer in SPP gas sensors with Kretschmann configuration [37].

In the following, we will review our work on the synthesis of sol-gel based nanomaterials to develop plasmonic sensors. The first part is focused on sol-gel gas sensors based on localized surface plasmons and the second on extended surface plasmons.

## 2. Sol-Gel Nanomaterials for Optical Sensors Based on Localized Plasmons

Gas sensors based on localized plasmons can be easily fabricated by including the plasmonic nanostructures (metal nanoparticles, nano-arrays, *etc.*) within a sol-gel thin film. There are two main strategies to develop such systems: (i) to include the precursor for the metal NPs within the sol-gel solution and rely on the nucleation of the NPs during the curing of the sol-gel film; or (ii) to synthesize the plasmonic structures separately and then embed them within the sol-gel matrix.

The first approach (*in-situ* formation) is obviously more direct and straightforward but does have a series of limitations, in particular regarding the control on the metal NPs size and shape, their composition and distribution across the sample, and also the curing temperature required to reduce the precursor and nucleate metallic NPs. The latter approach (*ex-situ*) provides much larger control on the nanoparticle size (uniform NPs with the desired size and standard deviation on the size below 10% can be routinely prepared), their shape (simple examples are spheres, rods, pyramids *etc.*), their composition (pure metals, alloys and core-shell structures) and their organization (randomly dispersed or precisely positioned within the sol-gel matrix). At the same time, the procedure to prepare such nanocomposites is more complex and time-consuming. Both approaches have been successfully applied to the fabrication of thin film-based plasmonic sensors, and in the following we will discuss them separately.

### 2.1. In SituFormation of Plasmonic Nanoparticles

**Figure 1:**
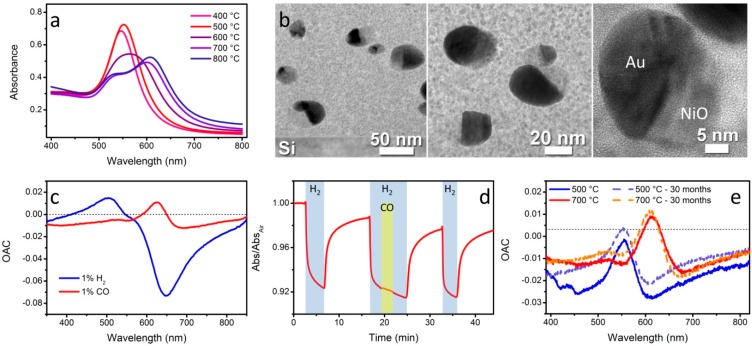
(**a**) Optical absorption spectra of SiO_2_-NiO-Au films annealed at different temperatures. The color of the spectra is representative of the actual color of the samples; (**b**) TEM images at different magnifications of SiO_2_-NiO-Au films showing the cookie-like nanostructures; (**c**) Optical absorption change (OAC) plot of SiO_2_-NiO-Au films exposed to hydrogen and CO at 300 °C showing the wavelength dependent response; (**d**) Time resolved tests of a SiO_2_-NiO-Au film at 300 °C showing selectivity for H_2_ even when the sensor is concurrently exposed to interfering CO (λ = 640 nm); (**e**) OAC plots of SiO_2_-NiO-Au films annealed either at 500 °C or 700 °C and then exposed to 1% CO at 300 °C a few days after being prepared and after 30 months from preparation.

Our first optical gas sensors based on localized surface plasmon resonance monitoring have been fabricated embedding NiO and Au NPs within a sol-gel SiO_2_ matrix. We initially developed a sol-gel protocol that enables the dispersion of functional oxide nanocrystals within a porous silica films at high concentration (up to 40%) without any aggregation or segregation phenomena that would be detrimental for the optical quality of the nanocomposites. Such films have been successfully used as optical and electrical gas sensors for the detection of carbon monoxide (CO), hydrogen (H_2_) and water vapors [38,39]. Building on these studies, we then modified the sol-gel recipe by adding a gold precursor and we were able to prepare SiO_2_-NiO-Au nanocomposites in which Au and NiO NPs are either separated or coupled according to the annealing temperature. Briefly, silicon alcoxides (tetraethoxysilane, TEOS and methyltriethoxysilane, MTES) dissolved in ethanol in the presence of water and hydrochloric acid are mixed with ethanolic solutions containing precursors for nickel (nickel chloride, NiCl_2_) and gold (tetrachloroauric acid, HAuCl_4_). A proper amino-functionalized silane is introduced to homogeneously disperse Ni^2+^ and Au^3+^ ions within the silica matrix. After depositing thin films via dip-coating, a thermal treatment (500–800 °C) is performed in order to fully condense and stabilize the silica matrix, and to nucleate NiO and Au nanoparticles. Notably, different thermal treatments cause different morphologies of the NiO-Au interface: at 500 °C physically separated, evenly dispersed Au and NiO NPs are observed, and the nanocomposite thin films show a sharp LSPR peak associated with spherical Au NPs embedded in an homogeneous matrix. When increasing the annealing temperature, a progressive red shift and shape change of the LSPR peak is observed (Figure 1a), which has been assigned to the formation of coupled Au/NiO heterostructures (named “cookie-like”) [40,41].

At high temperatures, the Au mobility is enhanced, and given the similarity in crystal structure between Au and NiO, the formation of epitaxially continuous Au/NiO hemispheres has been confirmed (Figure 1b). Within such heterostructures, Au is found to face NiO but also the SiO_2_ matrix: the two oxides have markedly different refractive indices, which is the reason why two separate LSPR contributions are observed.

The plasmonic features of such nanocomposites, together with their high active area provided by the porous sol-gel matrix, constitute a reliable and effective sensing platform to optically monitor reducing gases such as hydrogen and CO [42,43,44]. It is interesting to notice that Au-free SiO_2_-NiO nanocomposites have been proven to be able to optically detect reducing gases thanks to a variation in the absorption properties of NiO NPs. However, the presence of Au NPs generates a much more intense optical signal due to their distinctive LSPR feature. In addition, a wavelength dependent response is also achieved, which can eventually lead to selectively discriminate between different analytes if proper wavelength modulation analysis is used. In fact, as can be noticed from Figure 1c, different gases can generate different optical variations, which enable for the identification of spectral regions in which the material responds to only one target gas or reacts to both gases but with opposite response. This is easily visualized by plotting the Optical Absorption Change (OAC) parameter, defined as the difference between the absorption spectrum during target gas exposure and the absorption spectrum in air (OAC = A_Gas_ − A_Air_). Through a careful selection of the operative wavelength, real time monitoring of H_2_ and CO and selective discrimination between each other has been achieved (Figure 1d). Besides sensitivity and selectivity, long term stability is one of the main parameters that define a well-performing gas sensor: in this regard, we demonstrated how SiO_2_-NiO-Au films are still extremely sensitive to target gases after several months, even years (Figure 1e). Moreover, samples stabilized at higher temperatures (700 °C instead of 500 °C) show a much more stable response and a negligible variation after 30 months from preparation. All this confirms the viability of such sol-gel nanocomposites as active materials for plasmonic based gas sensors.

In a related study, we replaced Au with Ag using a different synthetic protocol which requires first the synthesis of a highly porous SiO_2_-NiO film, and then the impregnation of such films with Ag ions, followed by thermal reduction to form Ag NPs [45]. Since the LSPR frequency of Ag is different from that of Au, this material is responsive to reducing gases in a different wavelength range with respect to SiO_2_-NiO-Au, therefore broadening the operational parameters of such sensors.

Most of our research in optical sensors has been devoted towards the synthesis of nanomaterials for hydrogen and CO detection. However, we also investigated possible sensing materials for other important hazardous gases, such as hydrogen sulphide (H_2_S). To this extent, we developed a highly selective H_2_S sensor based on the optical monitoring of the LSPR variation of Au NPs dispersed within a NiTiO_3_ sol-gel matrix [46,47,48]. The sol-gel protocol adopted for such nanocomposites is similar to that developed for the SiO_2_-NiO-Au system, with the replacement of the Si precursors with titanium butoxide. Moreover, being titanium alcoxides much more reactive than the silicon equivalents, specific coordinating agents for Ti (such as acetylacetone) have to be added to prevent premature hydrolysis and condensation of the Ti-based network. Once again, the sol-gel matrix provides a porous network that allows the gas molecules to penetrate and reach the reactive sites, while Au NPs are responsible for the strong optical signal in the visible range associated with their LSPR peak. Interestingly, Au-free samples are not able to optically detect H_2_S; however, the nickel titanate based matrix has been proven able to successfully decompose hydrogen sulfide into sulfur oxides (SO_X_), with a yield comparable to NiTiO_3_-Au composites. 

**Figure 2:**
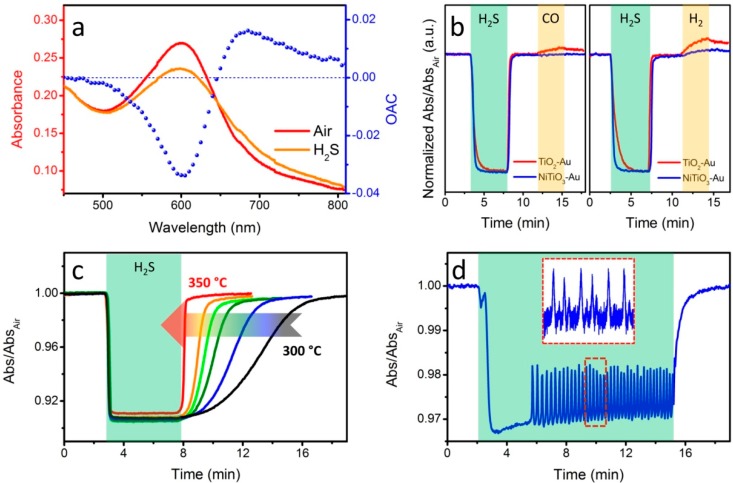
(**a**) Optical absorption spectra of a NiTiO_3_-Au film exposed to air and to 100 ppm H_2_S at 350 °C. The blue dotted plot (right vertical axis) shows the variation in absorbance as a function of the wavelength; (**b**) Time resolved tests for a NiTiO_3_-Au film showing the better response to H_2_S (100 ppm) and the absence of cross sensitivity to CO (1%) and H_2_ (1%) when compared to TiO_2_-Au film (T = 350 °C, λ = 605 nm); (**c**) Time resolved tests for a NiTiO_3_-Au film exposed to 100 ppm H_2_S showing the variation in recovery times as a function of the operating temperature (λ = 605 nm); (**d**) Oscillatory behavior of a NiTiO_3_-Au film exposed to 100 ppm H_2_S (T = 350 °C, λ = 590nm). The inset shows a zoomed view of a few oscillations.

This evidence suggests an active role of the oxide matrix in reacting with the target gas, while Au nanoparticles act as optical probes enabling optical detection. As shown in Figure 2a, a strong variation in optical absorption around the Au SPR peak is observed after exposing the sensing materials to H_2_S: the plasmon peak of Au is broadened and damped in the presence of hydrogen sulfide, suggesting a direct electronic interaction between sulfur and the free electrons at the surface of Au NPs. Minimal or no cross sensitivity towards interfering gases such as CO and H_2_ has been proved, especially after a careful selection of the operative wavelength used for the time-resolved tests. Moreover, increased selectivity for other reducing gases such as CO and H_2_ is achieved with respect to TiO_2_-Au nanocomposites. (Figure 2b). Such materials demonstrated very high sensitivity to H_2_S with detection limits below 10 ppm at operative temperatures between 300 and 350 °C, and very fast response times, of the order of 10–20 s. However, the recovery times are strongly affected by the temperature, as a consequence of the thermally activated desorption of sulfur species from the nanocomposite material. Given that the baseline is recovered in all tests performed above 300 °C (Figure 2c), we can conclude that the process is kinetically limited by such a desorption process.

A very distinctive feature of such nanomaterials is the ability to monitor chemical reactions occurring in real time. In fact, using plasmonic nanoparticles as optical probes, a chemical reaction affecting the plasmonic properties of the metal NPs (*i.e.*, their charge density, the refractive index or the chemical environment of the surrounding matrix, *etc.*) can be observed optically. To this extent, we discovered an interesting oscillatory behavior when our nanocomposites are exposed to hydrogen sulfide: a reversible, highly regular change in optical absorption is found to happen when specific samples were exposed to H_2_S (Figure 2d). Such evidence has been ascribed to a periodic change in local concentration of H_2_S and SO_X_ at the surface of the sensing material, generating small but regular variations in the LSPR feature of Au NPs [49]. Notably, the oscillatory behavior stops as soon as H_2_S flow is stopped and the gas sensing cell is flushed with the carrier gas (air). However, it can be re-observed when hydrogen sulfide is let flow again within the sensing cell, validating the claim that the optical oscillations are due to an interaction between the analyte and the sensing material. It is remarkable that such a simple plasmonic platform composed of metal nanoparticles embedded within a porous sol-gel matrix can be used not only for highly sensitive and selective gas sensors, but also as a tool for *in situ*, real-time monitoring of chemical reactions.

This approach is highly versatile and has been used as a proof-of-concept to prepare other metal oxides/Au composites (namely ZnO-Au and NiO-Au) with the sol gel technique, without using alcoxide precursors but relying on the hydrolysis and condensation of metal acetates/amine complexes in alcohols [50,51].

### 2.2. ExSituFabrication of Plasmonic Nanoparticles

It is well known that the optical properties of plasmonic nanoparticles depend on a variety of factors, including the size and shape of the NPs themselves and their relative proximity. Therefore, an accurate control over the formation and the positioning of plasmonic nanostructures within a sol-gel matrix becomes of vital importance when designing a highly performing optical gas sensor. For this reason, we developed several strategies to synthesize metallic NPs with highly controlled size and shape, which can be dispersed within a variety of sol-gel films. Moreover, we developed protocols to position metal nanostructures with controlled interparticle distance on a substrate that can eventually be covered with a sol-gel film.

We started to investigate a simple TiO_2_-Au system, in which Au spheres are synthesized with colloidal techniques, and after purification and surface functionalization, are dispersed within a titania-based sol-gel solution. Even if this is a simple binary system where Au NPs are embedded within a TiO_2_ matrix, there are some challenges that need to be addressed: for example, Au NPs have to be properly functionalized in order to enable for the preparation of highly concentrated and stable solutions which provide sufficient doping for the TiO_2_ matrix. Moreover, the colloidal stability of the Au NPs has to be maintained within the sol-gel solution, to avoid aggregation and precipitation of the NPs. We overcome these issues by developing a protocol in which Au NPs are synthesized with the desired size, and they are purified and concentrated in water using 11-mercaptoundecanoic acid (MUA): the thiol groups can strongly bind at the surface of Au, while the carboxylic acid moieties provide high solubility in water at basic pH. Alternatively, poly (N-vinylpyrrolidone) can be used as a ligand for Au NPs, providing good solubility and stability in ethanol. Such concentrated colloidal solutions can then be mixed with a sol-gel solution containing a Ti alcoxide and eventually deposited by spin coating and thermally annealed [52,53]. This method enables to control the nanoparticles size and shape within the TiO_2_ matrix, because the Au NPs are synthesized using tailored colloidal syntheses, and, therefore, we do not rely on the *in situ* reduction of metal salts during annealing of the TiO_2_ matrix. 

These nanocomposites have been used to detect reducing gases (H_2_, CO) at high operative temperatures (300–400 °C) [54], and VOCs (ethanol, methanol, isopropanol) at room temperature [53]. Reducing gases are found to cause a blue shift of the Au LSPR peak, causing a wavelength-dependent response as described in the previous section, while no optical response was observed for Au-free matrix. These nanocomposites have been successfully used to monitor real-time variation in the concentration of CO and H_2_ gas using either a plasmonic or a conductometric sensing platform, highlighting the versatility of such nanomaterials. Moreover, samples annealed at lower temperatures and used to detect VOCs can be doped with either Au spherical NPs or Au NRs, enabling a wider wavelength range of plasmonic response thanks to the red shift of the longitudinal plasmon resonance in metallic NRs. However, if such samples are annealed at temperatures greater than ~100 °C, the metal rods tend to become spheres in order to reduce their surface energy. Therefore, the range of operative temperatures of such nanocomposite is limited.

**Figure 3:**
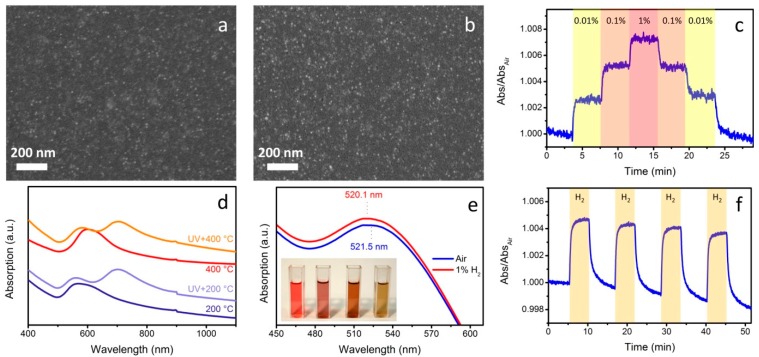
(**a**) SEM image of a TiO_2_-Au film annealed at 100 °C; (**b**) SEM image of a TiO_2_-Au film annealed at 500 °C; (**c**) Time resolved tests for a TiO_2_-Au film exposed to different concentrations of H_2_ (in volume %) at 300 °C (λ = 595 nm); (**d**) Optical absorption spectra of TiO_2_-Au NRs films synthesized from layered titanates showing the effect of UV treatment in avoiding the spheroidization of Au NRs; (**e**) Optical absorption spectra of TiO_2_ films containing Au-Pt core-shell NPs exposed to 1% hydrogen at room temperature. The inset shows Au-Pt colloidal solutions; (**f**) Time resolved tests for a TiO_2_-Au-Pt film exposed to 1% hydrogen at room temperature (λ = 500 nm).

An even greater degree of control in the microstructure and on the properties of the metal/metal oxide nanocomposites is achieved when also the TiO_2_ matrix is synthesized starting from colloidal NPs. Following this idea, we have developed an acid-catalyzed sol-gel protocol to synthesize small (~4 nm) anatase NPs that can be directly mixed with PVP-stabilized Au colloids and used to prepare TiO_2_-Au composite films [55]. Briefly, titanium isopropoxide is slowly added to a mixture of water, methanol and hydrochloric acid under vigorous stirring and mildly heated to nucleate small anatase crystals. The electrostatically stabilized colloids can be purified and redispersed in methanol at a high concentration suitable for thin film deposition via spin coating. Such suspensions are mixed with Au colloids in ethanol obtaining stable mixed colloidal solutions, thanks to the Au surface ligands which guarantee colloidal stability in solution and homogeneous dispersion of the metal NPs within the nanocrystalline TiO_2_ matrix (Figure 3a,b). This approach enables to prepare highly crystalline thin films at low temperatures, because there is no need to anneal at high temperature to trigger the crystallization of the titania sol-gel matrix, which has been found to occur above 400 °C. The very small TiO_2_ NPs also provide a larger surface area and an increased number of active sites with respect to traditional sol-gel films. Moreover, excellent surface contact between Au and TiO_2_ NPs has been demonstrated, and exploited to improve the gas sensing performances of these nanocomposites. In fact, reversible CO and hydrogen detection, with enhanced sensitivity compared to the state of the art TiO_2_-Au optical sensors has been achieved with our colloidal approach (Figure 3c). Detection limits below 10 ppm and response times as low as 10–20 s are demonstrated with nanocomposite films which are only 40–50 nm thick.

As discussed before, embedding already formed plasmonic NPs within a matrix allows unprecedented control on their size and shape: as such, we were able to dope anatase nanocrystalline films with very small (~3 nm) Au NPs, which show excellent reducing gases detection properties, and can provide some catalytic functionalities to the nanocomposites. In addition, room temperature optical detection of VOCs has also been achieved with these all-colloidal thin films [56]. In another study, we synthesized Au NRs with standard seeded-growth methods using cetyltrimethylammonium bromide (CTAB) and embedded them within a TiO_2_ nanocrystalline matrix: the challenge with this system is the incompatibility of the thermal stability window of Au NRs to avoid spheroidization (<100 °C) with the temperature required for efficient CO and H_2_ gas sensing measurements (>250 °C). We circumvent this issue by dispersing the NRs in a specifically designed TiO_2_-based matrix capable to undergo a substantial densification when exposed to UV light prior to the thermal annealing [57]. We synthesized layered titanates using a high temperature, base-catalyzed sol-gel method: titanium isopropoxide was first reacted with ethylene glycol at 110 °C, and then transformed into layered titanates upon reaction with tetramethylammonium hydroxide. After purification and resuspension in basic water, we could deposit thin films from such layered titanate inks. UV exposure is found to decompose the organic cations intercalated between the layers, causing the shrinking and densification of the inorganic matrix. Following thermal annealing at 400 °C, the titanates could be eventually transformed into crystalline anatase. The stiffness and improved density of the cured matrix enables to thermally anneal the nanocomposite up to 400 °C without detrimental effect on the Au NRs aspect ratio, which are enclosed within such rigid TiO_2_ matrix (Figure 3d). The use of Au NRs instead of spherical NPs allows the exploitation of both surface plasmon resonance frequencies (transversal and longitudinal) for plasmonic sensing of reducing gases, therefore increasing the operational tuneability compared to nanocomposites embedding Au spheres.

Unfortunately, all the sensing materials presented so far are rather insensitive to reducing gases at low operative temperatures. In fact, whilst VOCs can be detected at room temperature because they can affect the refractive index of the matrix surrounding the plasmonic nanoparticles, hydrogen and carbon monoxide require a certain activation temperature to react with the surface oxygen atoms of the metal oxide used as sensing material. We challenged and overcame this issue by developing plasmonic NPs in which an Au core is surrounded by a thin Pt shell. In this system, the Au core provides a strong LSPR peak which is used to monitor the target gas presence, while the platinum shell provides a highly catalytic surface to promote the target analyte reactions [58]. However, a Pt shell is also found to damp the surface plasmon resonance of Au NPs, limiting the plasmonic features useful for the optical detection of the target gas. For this reason, a very thin shell is preferred, because it provides enough catalytic activity without compromising the optical signatures of Au. We prepared TiO_2_-Au-Pt nanocomposites and demonstrated real-time hydrogen monitoring at room temperature with short transient times using surface plasmon spectroscopy: the presence of Au-Pt core-shell NPs within a sol-gel TiO_2_ colloidal matrix causes greater optical changes and faster detection with respect to TiO_2_-Au and TiO_2_-Pt nanocomposites (Figure 3e,f), enabling for the efficient optical detection of H_2_ at room temperature.

**Figure 4:**
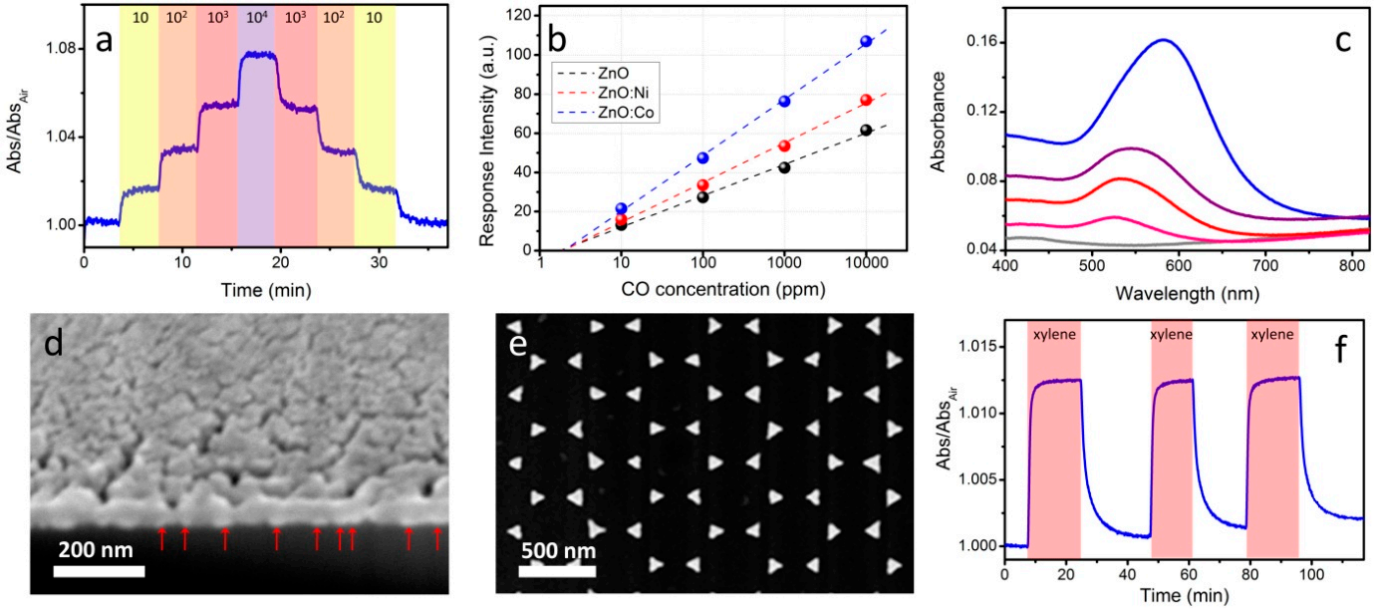
(**a**) Time resolved tests for a ZnO-Au film exposed to different concentrations of CO (in ppm) at 300 °C (λ = 570 nm); (**b**) Sensitivity plot for ZnO-Au films for CO detection according to the doping of ZnO NPs; (**c**) Optical absorption spectra of Au sub-monolayers with different surface coverage. The color of the spectra is representative of the actual color of the samples; (**d**) SEM image in cross section of a NiO film deposited on Au NP monolayer. The red arrows highlight the Au NPs at the NiO/substrate interface; (**e**) SEM image in top view of an Ag nanoprism array; (**f**) Time resolved measurements of an aryl-bridged polysilsesquioxane films deposited on top of an Ag nanoprism array when exposed to 30 ppm xylene at room temperature (λ = 653 nm).

The versatility of the *exsitu* approach has been also exploited to prepare ZnO-Au nanocomposites: a sol-gel synthesis based on the forced hydrolysis of zinc acetate has been used to synthesize ZnO colloidal NPs in ethanol/DMSO using tetramethylammonium hydroxide as a base/catalyst. Such NPs can be purified and concentrated in ethanol and eventually mixed with an ethanolic solution of PVP-capped Au NPs. The beauty of this approach is the possibility to independently synthesize and tune the properties of the prepared colloids: for example, we studied the effect of the doping of ZnO with different transition metal ions, analyzing their effect on the optical sensing properties of ZnO-Au nanocrystalline films monitoring the variation in the LSPR of Au NPs. A marked increase in sensitivity to CO has been observed when ZnO is doped with Co or Mn ions (Figure 4a,b), validating the strategy to synthesize high quality doped colloids and then using them in a nanocrystal ink to deposit functional nanocomposite films [59]. In a similar way, Au colloids can also be dispersed within a hybrid organic/inorganic sol-gel matrix bearing specific functionalities for the recognition of target molecules. We recently developed such nanocomposites using aryl-bridged polysilsesquioxane matrices which provide benzene rings suitable for recognition of aromatic molecules thanks to π-π coupling [60]. 

The LSPR feature of Au NPs was again used to monitor changes in the local environment surrounding the plasmonic centers (variation in refractive index, electronic density, *etc.*), while the sol-gel matrix provided a functional porous matrix capable of reacting reversibly with aromatic molecules such as xylene.

Another possibility offered by the *exsitu* synthesis of plasmonic nanoparticles, and their combination with sol-gel matrices relies first on the organization of the metal features on a substrate and on the subsequent deposition of sol-gel coatings. To this extent, we developed Au NP sub-monolayers where Au colloids are deposited on properly functionalized substrates, which enable for a strong chemical interaction between the functional moieties created on the substrate and those of the ligands surrounding Au NPs. In details, glass substrates have been functionalized with (3-aminopropyl) trimethoxysilane, a specific molecule bearing siloxane functionalities suitable for anchoring to the hydroxyl groups present at the surface of the glass slide, and an amine which can effectively react with MUA-capped Au NPs. In this way, stable Au sub-monolayers can be deposited with tunable surface coverage by simply changing the deposition conditions. Moreover, by modifying the surface coverage, the relative distance between the Au NPs can be tuned, therefore affecting the plasmonic coupling and the local field enhancement, which results in a red shift of the LSPR (Figure 4c) [61]. Such monolayers can be covered with a variety of sol-gel based matrices like oxides (TiO_2_, ZnO, NiO, see Figure 4d) or organic/inorganic hybrids and used for reducing gases or VOC detection [60,62,63]. The strategy described earlier (first deposition of metal features followed by the deposition of a sol-gel thin film) can be applied also to more complex architectures, where for example the metal features are nanoprism arrays fabricated by a combination of nanosphere lithography and thermal evaporation. In details, polystyrene nanospheres were self-assembled on a substrate and used as a mask for the thermal evaporation of a silver film. After removal of the mask with a simple lift off procedure using commercial tape, arrays of prisms with an approximately triangular base are obtained. Such ordered nanostructures provide multipolar resonances and local field enhancement at the vertices of the prisms that can be exploited for sensitive detection of gases and molecules (Figure 4e,f). For example, sub-ppm optical detection for aromatic hydrocarbons has been demonstrated when the nanoprism arrays are coated with sol-gel aryl-bridged polysilsesquioxane films [64].

## 3. Sol-Gel Nanomaterials for Optical Sensors Based on Extended Plasmons 

As discussed in the introduction, effective sensors for gases, vapors or liquids can be fabricated exploiting the optical signal of extended surface plasmons. Given the experience in nanomaterials and optical sensing available within our group, we investigated the possibility of using sol-gel chemistry to synthesize active materials for extended surface plasmon polaritons-based sensors.

We first started adopting a simple Kretschmann sensing platform in which a prism is used to couple the incident light beam and the surface plasmons on a flat metal surface in attenuated total reflection configuration. The active material is deposited as a thin layer on an Au/glass substrate and the sensing measurements are performed analyzing the intensity of the reflected light as a function of the incident angle and of the atmosphere the active material is exposed to. In such studies, the active material was either ZnO [65] or TiO_2_-Au [53]. The synthesis adopted for these thin films is similar to that described earlier; in details, ZnO is deposited from a sol-gel solution composed of zinc acetate dissolved in alcohol, possibly in the presence of ethanolamine, which helps dissolving the metal salt by coordinating the metal ions in solution. During the deposition via spin coating, the hydrolysis and condensation of metal complexes occur, and amorphous films are formed, that eventually crystallize following an annealing step. For TiO_2_ films, a solution of titanium isopropoxide in isopropanol is prepared, using acetic acid as a reactivity control agent to avoid fast and uncontrolled condensation of the alcoxide. Au NPs are added to this solution and films are deposited by spin coating and dried at 200 °C to ensure evaporation of volatile compounds and proper condensation of the inorganic titania matrix. Such films are exposed to vapors of alcohols (methanol, ethanol, isopropanol) and other VOCs (hexane) showing reversible sensing response at room temperature to all tested vapors. Moreover, the sensitivity is strongly dependent on the type of vapor, the type of sensing material, and the annealing temperature.

Recently, we further developed optical sensors based on extended plasmons by using a patternable sol-gel hybrid film to fabricate a dielectric-metal-dielectric (DMD) sinusoidal grating capable of coupling light and generating surface plasmon polaritons [28]. We used aryl-bridged polysilsesquioxane films similar to those described earlier thanks to their ability to undergo patterning and to their distinctive ability to detect aromatic hydrocarbons. Such films are deposited on glass substrate, patterned using a soft imprinting method using a silicone mould, and covered by a thin metal films using thermal evaporation. A second hybrid sol-gel film—the sensing layer—is deposited on top of the metal film to complete the DMD structure. Such device has been proven to detect down to 30 ppm xylene over a variety of different wavelengths corresponding to the different plasmonic dips detected in the reflection spectra, which are associated with extended plasmons. Even if the performances of this device architecture are not ideal, this study constitutes a proof of concept that sol-gel can be indeed used to fabricate functional materials and plasmonic devices thanks to its versatility and the ability to control film composition, porosity, thickness, functional groups and patterning.

## 4. Conclusions

Plasmonic gas sensors demonstrate great potential both for better understating the chemical interactions at the nanoscale and the development of practical optical sensors. Sol-gel nanostructured materials have been used as sensitive material in both LSPR and extended SPR based optical gas sensors. We demonstrated that through a careful control of the film structure, it is possible to achieve a very selective LSPR optical gas sensor.
